# A Systematic Literature Review of Injection Site Pain Perception in Adult Patients Treated with Citrate-Free and Citrate-Containing Biologic Agents

**DOI:** 10.2174/1573397118666220829123713

**Published:** 2023-06-05

**Authors:** Sophia Junker, Oliver Ebert, Robert Bartsch

**Affiliations:** 1 Ingress-Health HWM GmbH, a wholly owned subsidiary of Cytel Inc., Potsdamer Str. 58, 10785 Berlin, Germany;; 2 Amgen GmbH, Riesstraße 24, 80992 München, Germany

**Keywords:** Biologics, citrate, excipient, formulation, injection site pain, injection site reaction

## Abstract

**Objective:**

To investigate injection site pain (ISP) and other injection site outcomes caused by biologics administered alongside citrate-free (CF) and citrate-containing (CC) formulations.

**Methods:**

Electronic literature databases (Medline, Embase, and Cochrane Library) were systematically searched for clinical trials and observational studies reporting on injection site outcomes after subcutaneous administration of biologics. Studies with unknown excipient formulations were excluded. The primary outcome was ISP, and secondary outcomes included any other reported injection site reactions (ISRs). Meta-analysis approaches were used to aggregate evidence identified *via* the conducted systematic literature review.

**Results:**

A total of two observational studies, two cross-over/sequential trials, and three head-to-head comparison trials directly comparing CF with CC biologics were identified, as well as seven placebo-controlled trials. Evidence from five of the seven direct comparison studies suggested reduced pain perception at the injection site when CF formulations were applied. Findings for other ISRs were balanced between both formulations, with slightly favorable results for preparations without citrate. A meta-analysis of placebo-controlled trials found no significant difference between arms with CF formulations and placebo regarding the proportion of patients experiencing ISP (OR 0.62, 95% CI 0.30-1.28).

**Conclusion:**

Excipient formulations are rarely specified in studies assessing pain and other ISRs of subcutaneously administered biologics. The available data indicate that subcutaneous administration of biologic agents without citrate may be associated with lower pain perception outcomes compared with treatment using CC formulations. Importantly, ISP is influenced by many factors which may have affected the results. More research is needed to assess how formulation excipients influence ISRs.

## 1 INTRODUCTION

Biopharmaceutical agents, also known as biologics, have greatly influenced and enhanced treatment options for a variety of conditions. As opposed to compound generation *via* chemical synthesis, biologics are produced by genetically modified organisms *in vivo*, enabling the production of highly specific molecules such as human monoclonal antibodies [1]. A rapid gain in knowledge regarding the pathogenesis underlying immune-mediated inflammatory diseases (IMIDs) has facilitated the development of such targeted therapies for conditions such as rheumatoid arthritis (RA), psoriasis, and inflammatory bowel disease (IBD), including Crohn’s disease and ulcerative colitis [2-5]. As the development of biological proteins is highly complex, successor products, so-called biosimilars, underlie strict regulatory guidelines and must prove high similarity as well as non-inferiority to the reference product in terms of clinical efficacy and safety [6-8].

Biologics and biosimilars are usually administered *via* subcutaneous injection, intramuscular injection, or intravenous infusion [9]. The most common adverse events (AEs) of biologics are injection site reactions (ISRs), including erythema and/or itching, hemorrhage, related pain, and swelling [10-15]. These reactions occur across indications and may be influenced by various factors, including injection volume and needle size and sharpness [16, 17]. One factor which seems to greatly affect patient pain perception is the excipient formulation, *i.e*., the chemical composition of the buffer solution, which is administered alongside the biologic agent [18]. Pain as a side effect of subcutaneously administered biologics causes anxiety in affected patients and may thereby negatively impact the patients' quality of life, leading to a decrease in treatment adherence and compliance in some cases [18, 19]. Despite the requirements for approved biosimilars to be highly similar to the original biologic compounds themselves, the inactive ingredients of the injection formulation may differ. One crucial excipient which seems to increase perceived pain after subcutaneous injection is the compound citrate, which can be found in excipient formulations of biologics and biosimilars [18].

Even though evidence exists that citrate-free (CF) formulations may evoke less injection site pain (ISP) than buffers containing citrate, the certainty of this finding and the magnitude of this effect are unknown. Few previous studies summarizing the literature on injection site outcomes after subcutaneous injection with citrate-free/containing biologics have been published. Therefore, the main objective of this systematic literature review (SLR) was to summarize existing evidence regarding ISP and other injection site outcomes after subcutaneous administration of biologics with known formulations in terms of citrate content.

## 2 MATERIALS AND METHODS

This SLR was conducted in line with the Preferred Reporting Items for Systematic Reviews and Meta-Analyses (PRISMA) guidelines (http://www.prisma-statement.org). The protocol was not registered with a protocol registry and did not have a registration number.

### 2.1 Search Strategy and Selection Criteria

An automated electronic systematic literature search was conducted in Medline (*via* PubMed), EMBASE, and Cochrane Library^1^ during the time period Jan 1, 2009, up to Nov 18, 2019. Two separate algorithms were used to identify clinical trials and observational studies. The search string included treatment terms (identifying biologic or biosimilar treatments), endpoint terms (identifying ISP outcomes), and study design terms (identifying clinical trials or observational studies) in the English language. Identified studies were screened at the title, abstract, and full-text stage according to predefined population, intervention, comparison, outcome, and time frame (PICO-T) criteria. Included were retrospective and prospective observational real-world studies, clinical trials including both placebo-controlled trials and head-to-head comparisons, systematic reviews, and meta-analyses, which included patients >18 years of age with any observable indication receiving subcutaneously administered biologics or biosimilars and reported on ISP and/or other ISRs between 2009 and 2019. Publications reporting ISRs as adverse events were included. Studies were excluded from the final analysis, if one of the following applied: inclusion of patients <18 years of age; non-human study population; treatment other than subcutaneously administered biologics/biosimilars; case reports, nonsystematic reviews, editorials/letters; publication before January 1^st^, 2009. Reference lists of SLRs and meta-analyses were reviewed manually for the identification of additional relevant articles. In a final selection step, studies which did not specify the excipient formulation of their biologic treatments and/or did not report on a relevant comparison (biologic with CF formulation *versus* biologic with CC formulation or one of the specified formulations *versus* placebo) were excluded, unless consultation with a clinical expert led to unambiguous identification of unspecified treatment formulations. Two reviewers independently carried out the screening process; any conflicts were resolved after discussion with a third independent reviewer.

### 2.2 Data Extraction and Quality Assessment

Extraction items included general publication information, study design, patient baseline characteristics, and reported injection site outcomes. Primary outcomes were the percentage of patients experiencing ISP and the level of pain experienced by these patients. The percentages of patients experiencing other ISRs (separately per type of reaction) were included as secondary outcomes. Two reviewers independently carried out the data extraction of the identified studies; any conflicts were resolved after discussion with a third independent reviewer.

The scientific quality of eligible studies was assessed using the criteria recommended by the National Institute for Health and Care Excellence (NICE), the CONSORT (Consolidated Standards of Reporting Trials) 2010 for clinical trials and the STROBE (Strengthening the Reporting of Observational Studies in Epidemiology) guidelines as recommended by the Centre for Review and Dissemination (CRD) for observational studies [20-22]. The quality of each publication was summarized by the number of “yes” responses based on all eligible questions.

### 2.3 Meta-analysis Approach

A meta-analysis of placebo-controlled trials was conducted to assess whether ISP differed between biologics/biosimilars administered alongside citrate-free excipients and placebo injections. For this, available trial data on ISP after injection of CF biologics or placebo were aggregated in a Mantel-Haenszel fixed-effects model for binary data. Fixed effects models are used to calculate the weighted average of treatment effects estimated by individual studies. If a trial provided several treatment arms (all receiving CF biologics), the number of patients within the arms was combined for the comparison of CF biologic with placebo within the meta-analysis. Log-odds ratios were calculated based on the number of patients experiencing ISP after CF biologic/placebo injection. Statistical analyses were conducted using STATA (StataCorp. 2015. Stata Statistical Software: Release 14. College Station, TX: StataCorp LP).

## 3 RESULTS

### 3.1 Study Selection and Reported Outcomes

The initial electronic database search resulted in 2,305 initial hits after the removal of duplicates, including 412 observational studies and 1,893 clinical trials. Fourteen relevant studies were manually identified during the selection process. Screening according to prespecified PICO-T criteria yielded ten publications on observational studies and 24 publications on clinical trials. After the exclusion of articles not specifying the formulation in the final selection step, two observational studies (one full text of a study conducted in Japan and one conference abstract of a study conducted in Spain), as well as twelve clinical trials (ten full texts and two abstracts), were included in the SLR. Ten trials were multicountry studies, one trial was conducted in the USA, and one trial did not state the country. Fig. (**1**) provides the PRISMA flow charts showing the number of selected studies.

Included studies either recorded the level of pain at the injection site using the Visual Analog Scale (VAS), which ranges from 0 cm (or 0 mm; “No pain at all”) to 10 cm (or 100 mm; “Worst pain imaginable”), graded scales (*e.g*., low/moderate/high pain) and numerical scales (*e.g*., 1 “no pain” to 10 “highest pain”) or expressed the percentage of patients experiencing ISP. The percentage of patients experiencing other ISRs was often presented within the safety reporting.

### 3.2 Patient Population

Identified studies mainly included RA patients (n = 10), while some studies investigated patients with plaque psoriasis (PsO, n = 3), psoriasis (n = 2), Crohn’s disease (n = 2), psoriatic arthritis (PsA, n = 1), spondyloarthritis (SA, n = 1), spondylitis (n = 1), and IBD (n = 1). The mean age of the included patient groups ranged from 36.2 years to 61.5 years (reported for n = 10 studies). The proportion of females ranged between 26.0% and 94.9% in the included patient groups (n = 13), while 63-100% were of the white race (n = 6). On average, patient groups had been diagnosed with their respective disease 1.7-21.0 years ago (n = 11). Mean body mass index (BMI) ranged between 23.7 and 31.0 kg/m^2^ (n = 6). Studies reported 24.1-100.0% of the included patients to be biologic-naïve (n = 8). Characteristics of patient populations in each study arm are detailed in the supplement (Supplementary Table **1**).

### 3.3 Observational Studies: Outcomes

The two selected real-world studies included one prospective study and one retrospective chart review, with a total of 226 patients [23, 24]. Both studies directly compared a CC version of adalimumab with a CF formulation of the same agent and enrolled RA patients, while the prospective study also included patients with other indications. Details of the included observational studies are given in Table **1**.

The retrospective comparison study by Yoshida *et al.*, 2019 [24] surveyed 25 patients with RA and recorded pain perception on the VAS scale. At the time of injection, patients reported an overall average VAS score of 1.6 cm for the CF formulation and 6.7 cm for the CC formulation (mean difference: -5.1 cm, *p* <0.001). Ten minutes after injection, mean pain scores remained significantly lower in the CF group (CC: 3.1 cm *vs*. CF: 0.4 cm, mean difference: -2.7 cm, *p* < 0.001). More patients receiving the CC formulation reported any pain (CC: 100.0% *vs*. CF: 70.0%), while “no pain/almost no pain” was reported by more patients of the CF group (CC: 20.0% *vs*. CF: 80.0%). Additional ISRs were experienced by the CC group (redness 12.0% and swelling 8.0%), while no patients of the CF group reported these adverse events.

The prospective study of Martínez-Casanova *et al.*, 2019 [23] enrolled 201 patients of varying indications who had received both the previous CC adalimumab version and the newer CF version. Median VAS scores for the four investigated indications ranged from 4.0 cm to 6.0 cm with the CC formulation and from 0.0 cm to 2.0 cm with the CF formulation. The percentage of patients experiencing pain reduction when switching from CC to CF treatment ranged from 82.3% to 97.3%, with the biggest change in the IBD group.

### 3.4 Clinical Trials: Outcomes

Selected clinical trials included a total of 8,072 patients. The most common indication of enrolled patients was RA, and the most frequently analyzed agent was adalimumab. Two studies conducted direct crossover/sequential comparisons between the two formulations [25, 26] (Table **2**), while another three directly compared CF and CC excipient formulations in head-to-head comparisons [27-29] (Table **3**). One placebo-controlled trial compared a CF formulation and CC formulations with placebo [27], while seven other placebo-controlled trials analyzed pain-related outcomes for CF biologics and placebo only [30-36] (Supplementary Table **1**).

In the two randomized crossover studies reported by Nash *et al.*, 2016 [26], 125 RA patients rated pain perception during injection of a CF and a CC formulation of adalimumab. The two studies included in this publication reported a significantly lower immediate pain after injection of the CF formulation compared with the CC formulation (study 1, mean VAS: 3.3 cm CC *vs*. 1.6 cm CF; study 2, mean VAS: 4.2 cm CC *vs*. 0.9 cm CF; mean difference for pooled data: -2.5 cm, 95% CI -3.0 to -2.0, *p* < 0.001). More patients reported severe pain when receiving the CC formulation (CC: 13.9% *vs*. CF: 1.6%), while more patients reported mild pain when receiving the CF formulation (CC: 42.6% *vs*. CF: 86.9%). Fifteen minutes after injection, the present pain intensity associated with CF adalimumab injections remained significantly lower in study 2 (mean difference: -0.4 cm, 95% CI -0.6 to -0.2, *p* < 0.001), but not in study 1 (mean difference: -0.1, *p* = 0.581). No patients experienced ISRs when administered CC biologics; 3.3% of patients experienced unspecified ISRs when receiving the CF formulation in study 2.

In the open-label sequential trial by Muñoz *et al.*, 2018 [25], patients reported lower VAS scores for a CF adalimumab formulation given *via* auto-injection pen compared with a CC adalimumab formulation administered *via* prefilled syringe, with a difference of -3.0 cm (*p* < 0.001).

Three publications on head-to-head comparisons, including one publication describing two placebo-controlled phase-3 trials, directly compared the pain perception of patients receiving biologics administered using CF formulations with pain experienced by patients receiving CC formulations.

Krishnan *et al.*, 2018 [28] reported on two randomized, active controlled trials which compared a CF adalimumab biosimilar with a CC adalimumab reference: one study concerned RA patients and the other addressed PsO patients (total n = 876). For both indications, mean VAS scores were lower in the CF group compared with the reference group (CC: 16.1-21.4 mm *vs*. CF: 10.0-10.7 mm in RA patients; CC: 12.4-19.3 mm *vs*. CF: 3.3-4.5 mm in PsO patients). Additionally, slightly fewer ISRs were recorded for patients receiving adalimumab with the CF formulation compared with patients receiving the reference product (CC: 5.0% *vs*. CF: 2.3% in RA patients; CC: 5.2% *vs*. CF: 1.7% in PsO patients).

In a randomized phase 3 trial, Weinblatt *et al.*, 2013 [29] compared a CF formulation of abatacept with a CC adalimumab reference in 646 RA patients, both administered with background methotrexate (MTX). Overall, significantly fewer ISRs were reported in the abatacept group compared with the adalimumab group (CC: 9.1% *vs*. CF: 3.8%, 95% CI -9.1 to -1.6%, *p* = 0.006), where pain, erythema, pruritus, and unspecified ISRs were slightly more common in the CC group; hematoma was slightly more common in the CF group.

Griffiths *et al.*, 2015 [27] reported on two placebo-controlled trials which randomized a total of 2,562 PsO patients into groups receiving a CF formulation of etanercept, a CC formulation of ixekizumab (two different administration frequencies), or a placebo. In the pooled safety analysis, both treatments resulted in higher overall ISR rates compared with placebo (placebo: 4.0% *vs*. CC: 13.0%, 17.0% *vs*. CF: 16.0%). Rates of pain, erythema, and unspecified ISRs were similar between the intervention groups and 1.0% for the placebo group.

The other seven included placebo-controlled trials assessed the efficacy of a range of different agents with CF formulations [30-36]. In these publications, the percentage of patients experiencing ISP ranged from 0.0% to 4.9%, in comparison with 0.0% to 2.0% of patients assigned to a placebo group. Patients also experienced injection site erythema (ISE; CF: 0.0-7.0%; placebo: 0.0-2.0%) and unspecified ISRs (CF: 0.5-10.8%; placebo: 0.0-1.9%).

In studies directly comparing CF biologics with CC biologics, the most frequently reported outcomes were mean pain VAS scores as well as the percentages of patients experiencing pain, erythema, overall reactions, or unspecified reactions at the injection site. The available evidence on these outcomes is visually summarized in Fig. (**2**).

### 3.5 Meta-analysis

**Fig. (3):**
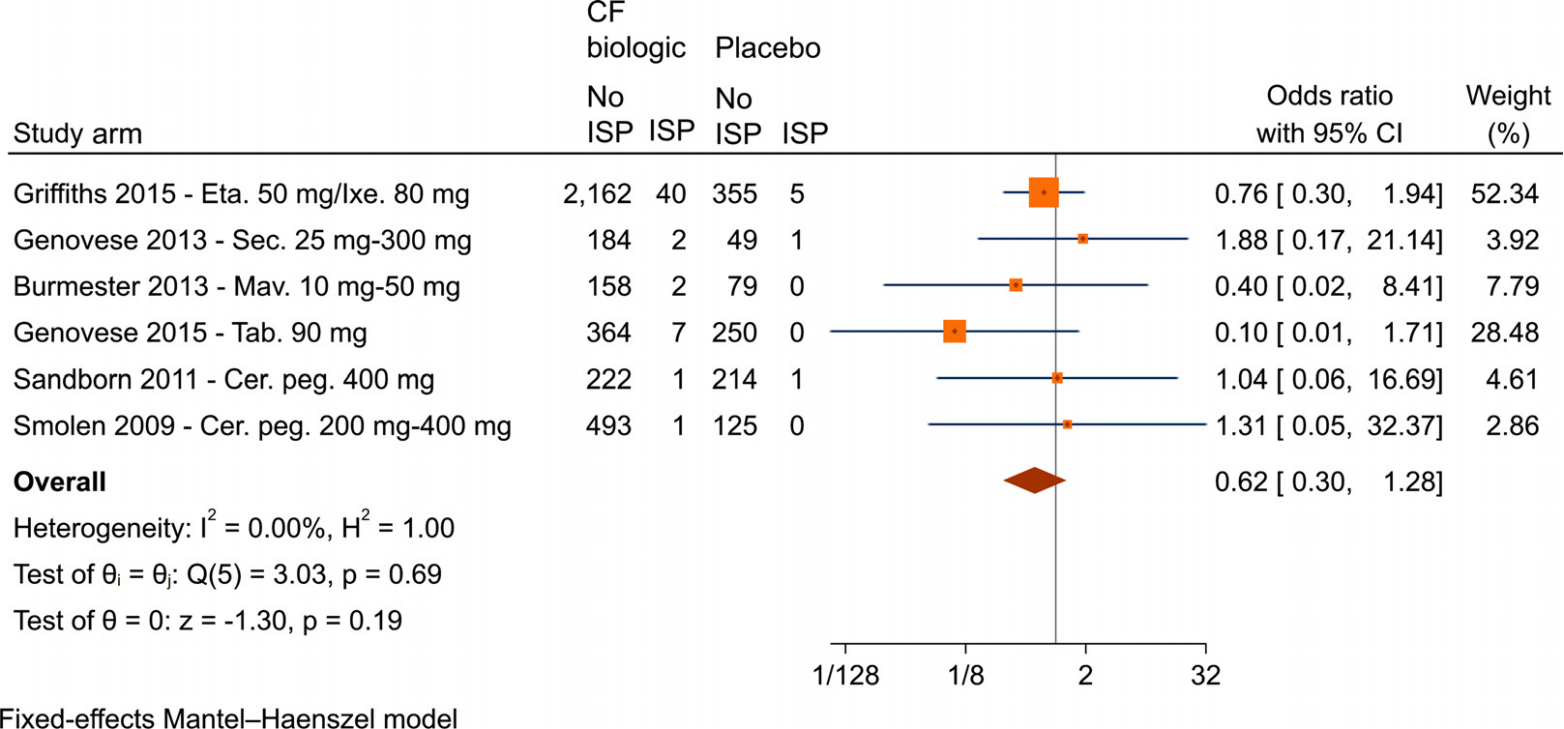
Aggregated results from placebo-controlled trials, comparing the number of patients experiencing injection site pain when administered biologics with citrate-free formulations *vs*. placebo. **Abbreviations:** Cer. peg., certolizumab pegol; CF, citrate-free; Eta, etanercept; ISP, injection site pain; Ixe, ixekizumab; Mav., mavrilimumab; Sec., secukinumab; Tab., tabalumab.

The data from randomized trials comparing placebo with CF biologics were aggregated regarding the percentage of patients experiencing pain at the injection site (Fig. **3**). Overall, the analysis suggested no significant difference in pain perception between biologics administered in conjunction with CF formulations compared with placebo injections (saline/sodium chloride solution or mixtures of amino acids and polysorbate with or without sorbitol), with an overall odds ratio of 0.62 (95% CI 0.30-1.28).

## 4 DISCUSSION

Within the scope of this SLR, we identified studies reporting pain and other injection site outcomes after subcutaneous injection of biologics and biosimilars of known formulations and evaluated whether the existence of citrate as a buffer excipient had an impact on perceived pain and other reactions at the injection site.

One important consideration for this review is the fact that formulation effects have been compared across a range of biologic agents and treated indications. Notably, in the majority of included studies comparing formulations which differ regarding the excipient citrate, patients were treated with the TNF-α inhibitor adalimumab or biosimilars of the latter [23-26, 28]. Conversely, a range of different agents was investigated in the included placebo-controlled trials, which reported on citrate presence within the intervention’s formulation.

Overall, outcomes in direct comparison studies were favorable for CF formulations in terms of perceived ISP. Both observational real-world studies included in this review showed lower ISP VAS scores for CF formulations of adalimumab compared with the reference product, one of which reported a significant difference. Additionally, the proportion of patients experiencing any pain after receiving a CC injection was reported as markedly higher than in the CF group. While all clinical trials with crossover or sequential trial design reported significantly lower pain scores for CF formulations, only one of the three direct head-to-head comparisons found similar findings, with the other two reporting similar pain rates in both comparison groups. Overall, differences regarding pain measured *via* VAS ranged between -2.5 cm and -5.1 cm (CC *vs*. CF). The difference in the percentage of patients experiencing any ISP in CF *vs*. CC groups also varied widely between studies (±0.0% to -97.3%). The rate of other ISRs was found to be balanced between CF and CC formulations in most included studies, except for one phase 3 trial reporting significantly lowered overall ISRs in the patient group receiving a CF formulation of abatacept.

In our analysis, we aggregated the results of RCTs comparing CF biologics by using a meta-analysis approach, and it could be shown that the percentage of patients who perceived pain at the injection site did not differ significantly between arms receiving CF biologics and the corresponding placebo arms.

More recently, Shi *et al*. specifically investigated the impact of buffer formulations in a randomized cross-over trial by applying different combinations of commonly used excipients without active ingredients [37]. The study found that a variety of formulation variables exert an impact on ISP. Shi and colleagues argue that it is not only the buffer type which plays a role in the extent of ISP, but the rather the combination of buffer type and concentration, ionic tonicity agent and concentration, and the solution’s pH. Similar to our findings, citrate-containing formulations, specifically those with high concentrations of citrate, were associated with increased ISP. A rising number of studies aimed at elucidating the underlying mechanism which explains the association of citrate with increased ISP. For example, Eaton *et al*. showed that commonly used citrate-containing buffer systems cause an increase in rodent dorsal root ganglia (DRG) firing, which is higher compared with that of other buffer systems containing saline or histidine, where the neuron firing represented a surrogate for nociception [38]. Another study by Yang *et al*. highlighted the role of acid-sensing ion channels (ASIC) in acid-related perception, showing that neutral citrate leads to the removal of the inhibitory effect of extracellular calcium ions on ASIC1 and thereby potentiates acid nociception in mice [39].

Despite the evidence found by Shi *et al*. [37] and biochemical pathways connecting citrate to ISP, the conclusion which can be drawn based on the evidence included in this review remains limited. This may partly be explained by the vast number of variables evidently associated with ISP which were not identifiable in all of the included studies and therefore could not be considered in the analysis. A recent review by Usach *et al*. summarized the evidence available on factors influencing ISP and recommended approaches to minimize pain at the injection site [40]. In addition to buffer, tonicity/osmolarity, and pH, the amount of injected volume, the site of injection, the injection speed, and needle features, such as size and sharpness, have been found to impact the extent to which the injection recipient experiences ISP.

## 5 LIMITATIONS

Most limitations of this review are related to between-trial differences in the study design, patient characteristics, and comedication, hampering immediate comparison. Studies differed regarding duration, follow-up time, administration frequency, and the time interval between injection and pain assessment, as well as reported injection site endpoints. Patient characteristics such as age, gender, and disease duration varied widely between studies. One of the most frequently reported endpoints was the percentage of patients experiencing unspecified reactions; however, the specific patient reactions were unknown, making it difficult to draw conclusions on these findings. Furthermore, it needs to be considered that within this SLR, the impact of CF and CC formulation on injection site outcomes was compared across treatments and indications. As the type of agent may exert an impact on pain and other ISRs, combining different agents and dosages in one comparison group for the aggregated analysis of placebo-controlled trials bears limitations. In addition, many factors which evidently play a role in ISP, such as needle features and injection speed, were only mentioned in a few of the included studies and therefore could not be considered in the analysis. Studies enrolled patients experiencing a wide variety of IMIDs, which may also have an impact on pain perception. Moreover, pain and other ISRs were reported as secondary outcomes in all included placebo-controlled trials. Finally, the number of participants in the included studies ranged between 25 and 2,562 patients, rendering some findings more meaningful than others. The variability of treatment regimens, study quality, definitions of outcomes, and the number of included patients limited our ability to directly compare the results of included studies.

Specific limitations exist regarding individual studies: In two of the studies comparing CF with CC versions of adalimumab, the CF formulation was administered using a smaller syringe and a smaller injection volume [23, 26]. This may have additionally influenced ISP perception as injection volume and needle size affect the development of reactions at the injection site [16, 17]. It also needs to be noted that in one study reporting lower VAS scores for a CF formulation, this treatment was administered *via* auto-injection pen while the CC formulation administered was given *via* a pre-filled syringe [25]. Furthermore, three of the investigated direct comparison publications are conference abstracts [23, 25, 28], impeding the investigation of study design and patient characteristics as well as the quality assessment of these studies.

## 6 STRENGTHS

This review gives a holistic overview of studies investigating pain perception and other injection site outcomes caused by biologics of known formulations in terms of citrate content. To the authors’ knowledge, this is the first study which systematically reviewed the existing literature regarding the mentioned study question. Findings from both clinical trials as well as observational studies have been investigated in a detailed manner, including results across a range of different indications. Hereby, we illustrate the range of different injection site outcomes which explain patients’ burden caused by subcutaneous injections across agents and indications.

In our study, head-to-head comparisons of CF and CC formulations provide the most robust evidence regarding injection site outcomes, even though only three of those studies could be identified. These trials each relied on a large sample size (>600 patients each). Where reported, no within-trial differences existed in terms of patient characteristics. Additionally, a meta-analysis based on a large total patient population (>6,300 patients) could be conducted to summarize results from placebo-controlled trials with CF arms.

## CONCLUSION

Formulations for subcutaneous injection of biologics which contain citrate may promote increased perceived pain at the injection site [18]. In our systematic review, only a few studies assessing ISP and other ISRs of biologics with known excipient formulations could be identified. Individual clinical studies and observational studies directly comparing the two formulations indicate that treatment with CF formulations of biologic agents may be associated with lower pain perception outcomes compared with treatment using CC formulations. However, the existing evidence is generally weak. On the other hand, a meta-analysis of aggregated data from placebo-controlled trials showed no significant difference in pain perception between CF injections and placebo, suggesting that injections without citrate may not cause more pain at the injection site than sham injections. Although this analysis was based on a large overall patient population, the findings have been compared across biologic intervention agents and indications, which may exert an influence on pain perception and other injection site outcomes. Importantly, many unmeasured factors contribute to the extent of perceived pain at the injection site, which may have influenced the results. As pain caused by medication injection leads to poor tolerability and anxiety in treated patients and negatively affects treatment adherence [18, 19], further research to identify factors influencing this outcome is needed.

## Figures and Tables

**Fig. (1):**
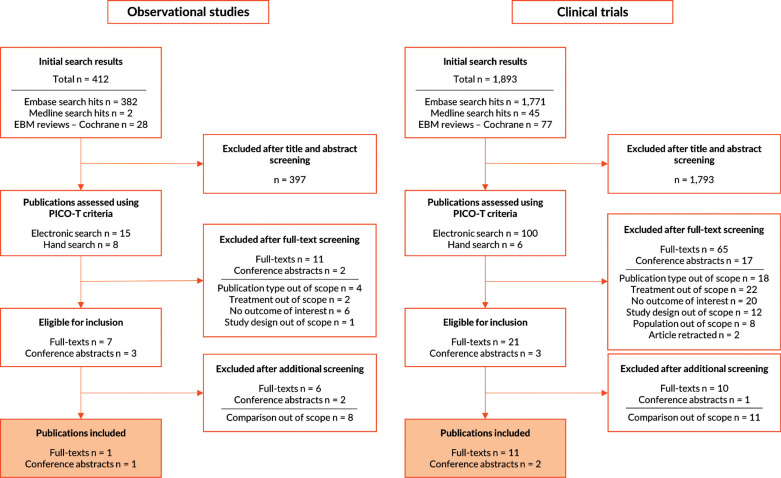
Selection of publications included in the systematic review.

**Fig. (2):**
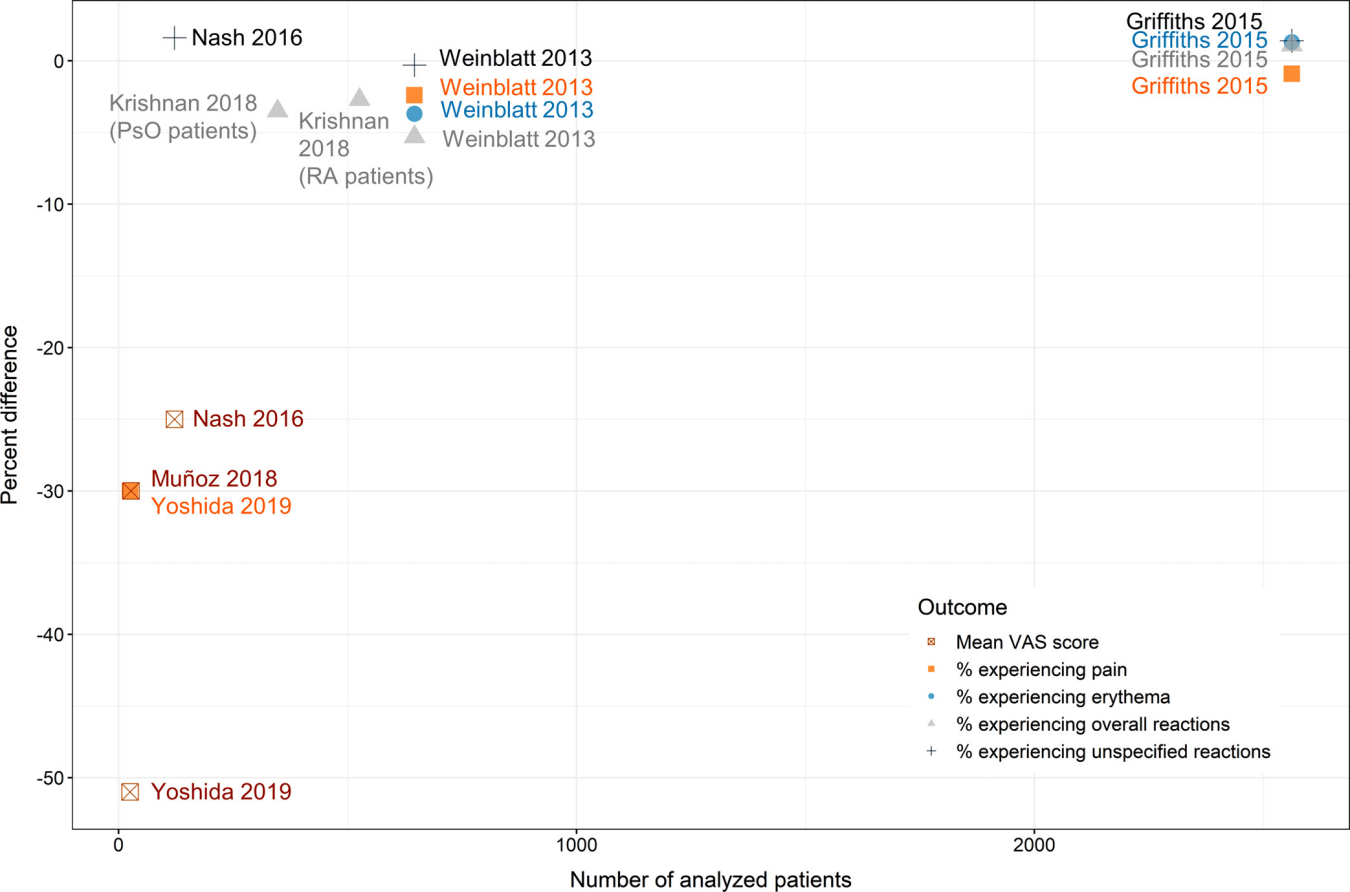
Percent difference in injection site outcomes between biologics administered with CC *vs*. CF formulations reported in included head-to-head comparisons, cross-over trials, and observational studies. A Lower per cent difference corresponds to a lower score or % of patients experiencing a certain outcome in the respective CF arm. For the purpose of visualization, only the first author and year of the corresponding publication are depicted.

**Table 1:** Observational studies comparing ISP intensity and other ISRs between biologics administered using CF and CC formulations.

**References**	**Article Type**	**Study Type**	**Country**	**Sample Size**	**Indication**	**Interventions and Formulations**	**Clinical Outcomes at Injection Site**	**ISP: Key Results**	**ISRs: Key Results**	**Key Conclusion**	**Outcome ISP**	**Outcome ISRs**
Yoshida *et al.* 2019 [24]	Full text	Retrospec- tive study based on patient survey	Japan	25	RA	Adalimumab (CC) Adalimumab (CF)	Mean pain VAS score % swelling % redness	VAS score CC: 6.7 cm; CF: 1.6 cm Diff: -5.1 cm, p < 0.001 Pain CC: 100%; CF: 70%	Redness CC: 12%; CF: 0% Swelling CC: 8%; CF: 0%	The new citrate-free adalimumab formulation caused less perceived pain compared with the citrate-containing reference product.	Signif. positive for CF	Positive for CF
Martinez-Casanova *et al.* 2019 [23]	Abs- tract	Prospective study performed during the adalimumab formulation shift in 2017	Spain	201	RA, SA, IBD, psori- asis	Adalimumab (CF) Adalimumab (CC)	Median pain VAS score (% diff)	VAS score CC: 6 cm (RA), 6 cm (SA), 6 cm (IBD), 4 cm (psoriasis); CF: 2 cm (RA), 0 cm (SA), 0 cm (IBD), 0 cm (psoriasis) Diff: 82.3% (RA), 88.7% (SA), 97.3% (IBD), 83.3% (psoriasis)	-	Lower pain scores were observed for the citrate-free formulation of adalimumab across indications and a high percentage of patients experienced an ISP reduction when switching from the CC to the CF formulation.	Positive for CF	NA

**Abbreviations:** CC, citrate-containing; CF, citrate-free; ISP, injection site pain; ISR, injection site reaction; IBD, inflammatory bowel disease; RA, rheumatoid arthritis; SA, spondyloarthritis; VAS, Visual Analogue Scale. Significant results stated as reported in the respective publication; results were interpreted as balanced if the percent difference between CF and CC formulation outcomes was <5.0%.

**Table 2:** Crossover/sequential trials comparing ISP intensity and other ISRs between biologics administered using CF and CC formulations.

**Refer-** **ences**	**Article Type**	**Study Design**	**Country**	**Sample Size**	**Indication**	**Interventions and Formulations**	**Clinical Outcomes at Injection Site**	**ISP: Key Results**	**ISRs: Key Results**	**Key Conclusion**	**Outcome ISP**	**Outcome ISRs**
Nash *et al.* 2016 [26]	Full text	Two randomi- zed, single-blind, two period crossover studies	Multi-country	125	RA	Adalimumab (CC) Adalimumab (CF)	Mean pain VAS score % mild/severe pain % unspecified reactions	Pooled VAS score CC: 3.7 cm; CF: 1.2 cm; Diff: -2.5 cm, 95% CI -3.0 to -2.0, p < 0.001 Mild pain (pooled) CC: 42.6%; CF: 86.9% Severe pain (pooled) CC: 13.9%; CF: 1.6%	Unspecified reactions CC: 0.0% (study 1), 0.0% (study 2) CF: 0.0% (study 1), 3.3% (study 2)	The citrate-free adalimumab formulation was well tolerated and associated with less injection site pain than the citrate-containing adalimumab formulation.	Signif. positive for CF	Balanced
Muñoz *et al.* 2018 [25]	Abstract	Open-label, single-arm, sequential trial	Un-known	27	Psoriasis, spondylitis, Crohn’s disease, PsA	Adalimumab (CC) Adalimumab (CF)	Mean pain VAS score	VAS score Diff: −3.0 cm, 95% CI -4.2 to −1.9, p<0.001		The auto-injection pen with citrate-free buffer was reported as superior to pre-filled syringe with citrate-containing buffer in terms of perceived injection site pain intensity.	Signif. positive for CF	NA

**Abbreviations:** CC, citrate-containing; CF, citrate-free; ISE, injection site erythema; ISP, injection site pain; ISR, injection site reaction; PsA, psoriatic arthritis; RA, rheumatoid arthritis; VAS, Visual Analogue Scale. Significant results were stated as reported in the respective publication; results were interpreted as balanced if the per cent difference between CF and CC formulation outcomes was <5.0%.

**Table 3:** Head-to-head comparison trials regarding ISP intensity and other ISRs between biologics administered using CF and CC formulations.

**Reference**	**Article Type**	**Study Design**	**Country**	**Sample Size**	**Indication**	**Biologic Intervention(s) and Formulations**	**Clinical Outcomes at Injection Site**	**ISP: Key Results**	**ISRs: Key Results**	**Key Conclusion**	**Outcome ISP**	**Outcome ISRs**
Krishnan *et al.* 2018 [28]	Abstract	Two randomized, double-blind, active-controlled clinical trials	USA	876	RA; PsO	Adalimumab (CC) Adalimumab (CF)	Mean pain VAS score % overall reactions	VAS score RA patients: CC: 16.1-21.4 mm; CF: 10.0-10.7 mm PsO patients: CC: 12.4-19.3 mm; CF: 3.3-4.5 mm	Overall reactions RA patients: CC: 5.0%; CF: 2.3% PsO patients: CC: 5.2%; CF: 1.7%	Frequency of ISRs and perception of ISP were lower with the CF biosimilar compared with the CC adalimumab reference.	Positive for CF	Balanced
Weinblatt *et al.* 2013 [29]	Full text	Multinational, prospective, randomized, Phase 3	Multi-country	646	RA	Adalimumab + MTX (CC) Abatacept + MTX (CF)	% pain % overall reactions % erythema % pruritus % hematoma % unspecified reactions	Pain CC: 2.4%; CF: 0.0%	Overall reactions CC: 9.1%; CF: 3.8% Erythema CC: 4.3%; CF: 0.6% Pruritus CC: 2.1%; CF: 0.3% Hematoma CC: 0.9%; CF: 1.6% Unspecified reactions CC: 1.2%; CF: 0.9%	Significantly more local overall ISRs occurred in patients treated with the CC adalimumab formulation.	Balanced	Signif. positive for CF
Griffiths *et al.* 2015 [27]	Full text	Two double-blind, multicenter, placebo-controlled Phase 3 trials	Multi-country	2,562	Chronic PsO	Ixekizumab Q2W (CC) Ixekizumab Q4W (CC) Etanercept (CF)	% pain % erythema % unspecified reactions	Pain CC: 3% (Q2W), 1% (Q4W); CF: 1%; Placebo: 1%	Overall reactions CC: 17% (Q2W), 13% (Q4W); CF: 16%; Placebo: 4% Erythema CC: 3% (Q2W), 12% (Q4W); CF: 4%; Placebo: 1% Unspecified reactions CC: 10% (Q2W), 9% (Q4W); CF: 11%; Placebo: 1%	Although injection site reactions were among the most common adverse reactions recorded after receiving ixekizumab, occurrences were similar to those in patients given etanercept and were mild or moderate in severity.	Balanced	Balanced

**Abbreviations:** CC, citrate-containing; CF, citrate-free; ISE, injection site erythema; ISP, injection site pain; ISR, injection site reaction; MTX, methotrexate; PsO, plaque psoriasis; RA, rheumatoid arthritis; VAS, Visual Analogue Scale. Significant results were stated as reported in the respective publication; results were interpreted as balanced if the per cent difference between CF and CC formulation outcomes was <5.0%.

## LIST OF ABBREVIATIONS

AE: Adverse Event
BMI: Body Mass Index
CC: Citrate-Containing
CF: Citrate-Free
CONSORT: Consolidated Standards of Reporting Trials
CRD: Centre for Review and Dissemination
IBD: Inflammatory Bowel Disease
IMID: Immune-Mediated Inflammatory Disease
ISE: Injection-Site Erythema
ISP: Injection-Site Pain
ISR: Injection-Site Reaction
MTX: Methotrexate
NICE: National Institute for Health and Care Excellence
NMA: Network-Meta Analysis
PICO-T: Population, Intervention, Comparison, Outcome, and Time Frame
PRISMA: Preferred Reporting Items for Systematic Reviews and Meta-Analyses
PsA: Psoriatic Arthritis
PsO: Plaque Psoriasis
Q2W: Once Every Two Weeks
Q4W: Once Every Four Weeks
QM: Once Every Month
RA: Rheumatoid Arthritis
RCT: Randomized Controlled Trial
SA: Spondylarthritis
SLR: Systematic Literature Review
STROBE: Strengthening the Reporting of Observational Studies in Epidemiology
TNF-α: Tumor Necrosis Factor Alpha
VAS: Visual Analog Scale

## SUPPLEMENTARY MATERIAL

The tables and PRISMA checklist are available as supplementary material on the publisher’s website along with the published article.

## NOTES

^1^ Cochrane Library includes the following databases: ACP Journal Club, Cochrane Central Register of Controlled Trials, Cochrane Clinical Answers, Cochrane Database of Systematic Reviews, Cochrane Methodology Register, Database of Abstracts of Reviews of Effects, Health Technology Assessment, NHS Economic Evaluation Database.
