# Supplementary material for: A Systematic Literature Review of Injection Site Pain Perception in Adult Patients Treated with Citrate-Free and Citrate-Containing Biologic Agents
Source: Curr Rheumatol Rev. 2023 Jun 5;19(3):303–13. doi: 10.2174/1573397118666220829123713 (PMC10433360; doi:10.2174/1573397118666220829123713)
Supplement: Supplementary file 1 [file CRR-19-303_SD1.pdf]

Supplementary Material

A Systematic Literature Review of Injection Site Pain Perception in Adult Patients Treated with Citrate-Free and Citrate-Containing Biologic Agents

Sophia Junker[1,*], Oliver Ebert[2] and Robert Bartsch[2]

[1]*Ingress-Health HWM GmbH, a wholly owned subsidiary of Cytel Inc., Potsdamer Str. 58, 10785 Berlin, Germany;* [2]*Amgen GmbH, Riesstraße 24, 80992 München, Germany*

Supplementary Table 1. Characteristics of study populations.

Reference	Type of study	Disease	Intervention(s)		Sample size	Mean age (years)	Female (%)	Race white (%)	BMI (kg/m²)	Disease duration (years)	Biologic-naïve (%)
Yoshida *et al.* 2019 [24]	Observational study	RA	Adalimumab (CC) & Adalimumab (CF)		25	61.5	80.0	-	-	13.1	65.0
Martinez-Casanova *et al.* 2019 [23]	Observational study	RA	Adalimumab (CC) & Adalimumab (CF)		34	61.2	62.0	-	-	-	-
		SA			106	52.6	39.0	-	-	-	-
		IBD			37	44.7	35.0	-	-	-	-
		Psoriasis			24	50.9	38.0	-	-	-	-
Nash *et al.* 2016 [26]	Cross-over trial	RA	Adalimumab (CC) -> Adalimumab (CF)	Study 1	31	58.6	67.7	-	-	16.8	29.0
				Study 2	29	54.4	79.3	-	-	12.1	24.1
			Adalimumab (CF) -> Adalimumab (CC)	Study 1	31	51.1	67.7	-	-	9.3	32.2
				Study 2	31	58.3	77.4	-	-	11.7	32.3
Muñoz *et al.* 2018 [25]	Sequential trial	Psoriasis, spondylitis, Crohn's disease, PsA	Adalimumab (CC) & Adalimumab (CF)		27	-	32.3	-	-	-	-
Krishnan *et al.* 2018 [28]	Head-to-head comparison	RA	Adalimumab (CC)		262	-	-	-	-	-	-
		PsO			173	-	-	-	-	-	-
		RA	Adalimumab (CF)		264	-	-	-	-	-	-
		PsO			174	-	-	-	-	-	-
Weinblatt *et al.* 2013 [29]	Head-to-head comparison	RA	Adalimumab (CC)		328	51.0	82.3	78.0	-	1.7	100.0
			Abatacept (CF)		318	51.4	81.4	80.8	-	1.9	100.0
Griffiths *et al.* 2015 [27]	Placebo-controlled head-to-head comparison	Chronic PsO	Placebo	Study 1	168	45.0	29.0	89.0	31.0	19.0	74.0
				Study 2	193	46.0	29.0	91.0	30.0	18.0	83 .0
			Ixekizumab Q4W (CC)	Study 1	347	45.0	30.0	92.0	31.0	19.0	75.0
				Study 2	386	46.0	33.0	93.0	31.0	18.0	85.0
			Ixekizumab Q2W (CC)	Study 1	351	45.0	37.0	94.0	30.0	18.0	76.0
				Study 2	385	46.0	34.0	94.0	30.0	18.0	85.0
			Etanercept (CF)	Study 1	358	45.0	34.0	94.0	31.0	19.0	79.0
				Study 2	382	46.0	30.0	92.0	31.0	18.0	84.0
Genovese *et al.* 2013 [31]	Placebo-controlled trial	RA	Placebo		50	55.0	68.0	-	27.5	6.8	77.8
			Secukinumab 25 mg (CF)		54	53.3	83.3	-	26.7	6.7	81.6
			Secukinumab 75 mg (CF)		49	54.3	77.6	-	27.0	8.6	79.1
			Secukinumab 150 mg (CF)		41	57.8	81.4	-	27.4	7.9	80.5
			Secukinumab 300 mg (CF)		50	54.7	75.6	-	29.5	5.9	80.0
Burmester *et*	Placebo-controlled	RA	Placebo		75	50.9	88.0	100.0	26.1	7.5	94.7

1875-6360/23 $65.00+.00 © 2023 Bentham Science Publishers

Reference	Type of study	Disease	Intervention(s)	Sample size	Mean age (years)	Female (%)	Race white (%)	BMI (kg/m^2)	Disease duration (years)	Biologic-naïve (%)
al. 2013	trial		Mavrilimumab 10 mg (CF)	39	52.2	82.1	100.0	25.1	9.8	82.1
			Mavrilimumab 30 mg (CF)	41	49.7	87.8	92.7	26.6	5.6	97.6
			Mavrilimumab 50 mg (CF)	39	53.3	94.9	100.0	26.1	7.5	100.0
			Mavrilimumab 100 mg (CF)	39	49.3	87.2	100.0	26.9	6.4	100.0
Genovese *et al.* 2015 [32]	Placebo-controlled trial	RA	Placebo	251	51.0	83.0	65.0	27.4	5.8	-
			Tabalumab 90 mg (CF)	374	50.6	79.0	63.0	27.5	5.7	-
			Tabalumab 120 mg (CF)	379	52.4	77.0	67.0	27.1	6.2	
Papp *et al.* 2016 [33]	Placebo-controlled trial	PsO	Placebo	220	47.0	27.0	92.0	30.3	21.0	54.0
			Brodalumab 140 mg (CF)	219	46.0	26.0	90.0	30.5	19.0	55.0
			Brodalumab 210 mg (CF)	222	46.0	27.0	91.0	31.0	20.0	53.0
Sandborn *et al.* 2011 [34]	Placebo-controlled trial	Crohn's disease	Placebo	215	38.8	58.0	-	23.7	7.0	-
			Certolizumab pegol (CF)	223	36.3	52.9	-	24.9	7.5	-
Smolen *et al.* 2009 [35]	Placebo-controlled trial	RA	Placebo + MTX	127	51.5	84.3	-	-	5.6	-
			Certolizumab pegol 200 mg + MTX (CF)	246	52.2	83.7	-	-	6.1	-
			Certolizumab pegol 400 mg + MTX (CF)	246	51.9	78.0	-	-	6.5	-
Emery *et al.* 2009 [36]	Placebo-controlled trial	RA	Placebo + MTX	160	48.6	83.8	71.3	-	2.9	100.0
			Golimumab 50 mg + MTX (CF)	159	50.9	84.9	74.8	-	3.5	100.0
			Golimumab 100 mg (CF)	159	48.2	84.3	69.8	-	4.1	100.0
			Golimumab 100 mg + MTX (CF)	159	50.2	78.6	73.6	-	3.6	100.0

CF, citrate-free; MTX, methotrexate; IBD, inflammatory bowel disease; PsA, psoriatic arthritis; PsO, plaque psoriasis; RA, rheumatoid arthritis

Supplementary Table 2. Placebo-controlled trials investigating ISP intensity and/or other ISRs of biologics administered using CF formulations.

.	Article type	Study design	Countries	Sample size	Indication	Interventions and dosages	Clinical outcomes at injection site	ISP: Key results	ISR: Key results
Genovese *et al.* 2013 [31]	Full text	Multicenter, dose-finding, double-blind, randomized, Phase 2	Multi-country	237	RA	Secukinumab 25 mg QM<br>Secukinumab 75 mg QM<br>Secukinumab 150 mg QM<br>Secukinumab 300 mg QM	% pain<br>% erythema	Pain<br>CF: 0.0%, 2.0%, 0.0%, 4.9%<br>Placebo: 2.0%	Erythema<br>CF: 1.9%, 2.0%, 4.7%, 2.4%<br>Placebo: 2.0%
Burmester *et al.* 2013 [30]	Full text	Multicenter, randomized, double-blind placebo-controlled, Phase 2	Multi-country	239	RA	Mavrilimumab 10 mg Q2W + MTX<br>Mavrilimumab 30 mg Q2W + MTX<br>Mavrilimumab 50 mg Q2W + MTX<br>Mavrilimumab 100 mg Q2W + MTX	% pain	Pain<br>CF: 0.0%, 2.4%, 0.0%, 2.5%<br>Placebo: 0.0%	-
Genovese *et al.* 2015 [32]	Full text	Multicenter, randomized, placebo-controlled, double-blind, Phase 3	Multi-country	1,004	RA	Tabalumab 90 mg Q2W<br>Tabalumab 120 mg Q4W	% pain	Pain<br>CF: 2.0%, NA<br>Placebo: 0.0%	-
Papp *et al.* 2016 [33]	Full text	Double-blind, placebo-controlled, Phase 3	Multi-country	661	PsO	Brodalumab 140 mg Q2W<br>Brodalumab 210 mg Q2W	% overall reactions	-	Overall ISRs<br>CF: 0.5%, 1.4%<br>Placebo: 0.0%
Sandborn *et al.* 2011 [34]	Full text	Multicenter, randomized, double-blind, placebo controlled	Multi-country	439	Crohn's disease	Certolizumab pegol 400 mg Q2W	% pain<br>% unspecified reactions	Pain<br>CF: 0.4%<br>Placebo: 0.5%	Unspecified ISR<br>CF: 4.9%<br>Placebo: 0.5%
Smolen *et al.* 2009 [35]	Full text	Multicenter, randomized, double-blind, placebo-controlled, Phase 3	Multi-country	619	RA	Certolizumab pegol 200 mg Q2W + MTX<br>Certolizumab pegol 400 mg + MTX	% pain<br>% unspecified reactions	Pain<br>CF: 0.0%, 0.4%<br>Placebo: 0.0%	Unspecified ISRs<br>CF: 1.2%, 2.0%<br>Placebo: 0.0%
Emery *et al.* 2009 [36]	Full text	Randomized, double-blind, placebo-controlled, Phase 3	Multi-country	637	RA	Golimumab 50 mg Q4W + MTX<br>Golimumab 100 mg Q4W<br>Golimumab 100 mg Q4W + MTX	% erythema<br>% unspecified reactions	-	Erythema<br>CF: 5.1%, 7.0%, 5.7%<br>Placebo: 0.0%<br>Unspecified ISRs<br>CF: 4.4%, 10.8%, 8.8%<br>Placebo: 1.9%

CF, citrate-free; MTX, methotrexate; ISP, injection site pain; ISR, injection site reaction PsO, plaque psoriasis; RA, rheumatoid arthritis.

Section and Topic	Item #	Checklist item	Location where item is reported
**TITLE**			
Title	1	Identify the report as a systematic review.	1
**ABSTRACT**			
Abstract	2	See the PRISMA 2020 for Abstracts checklist.	2-3
**INTRODUCTION**			
Rationale	3	Describe the rationale for the review in the context of existing knowledge.	4-5
Objectives	4	Provide an explicit statement of the objective(s) or question(s) the review addresses.	4.5
**METHODS**			
Eligibility criteria	5	Specify the inclusion and exclusion criteria for the review and how studies were grouped for the syntheses.	6
Information sources	6	Specify all databases, registers, websites, organisations, reference lists and other sources searched or consulted to identify studies. Specify the date when each source was last searched or consulted.	6
Search strategy	7	Present the full search strategies for all databases, registers and websites, including any filters and limits used.	6
Selection process	8	Specify the methods used to decide whether a study met the inclusion criteria of the review, including how many reviewers screened each record and each report retrieved, whether they worked independently, and if applicable, details of automation tools used in the process.	6-7
Data collection process	9	Specify the methods used to collect data from reports, including how many reviewers collected data from each report, whether they worked independently, any processes for obtaining or confirming data from study investigators, and if applicable, details of automation tools used in the process.	6-7
Data items	10a	List and define all outcomes for which data were sought. Specify whether all results that were compatible with each outcome domain in each study were sought (e.g. for all measures, time points, analyses), and if not, the methods used to decide which results to collect.	7
	10b	List and define all other variables for which data were sought (e.g. participant and intervention characteristics, funding sources). Describe any assumptions made about any missing or unclear information.	7
Study risk of bias assessment	11	Specify the methods used to assess risk of bias in the included studies, including details of the tool(s) used, how many reviewers assessed each study and whether they worked independently, and if applicable, details of automation tools used in the process.	7
Effect measures	12	Specify for each outcome the effect measure(s) (e.g. risk ratio, mean difference) used in the synthesis or presentation of results.	7
Synthesis methods	13a	Describe the processes used to decide which studies were eligible for each synthesis (e.g. tabulating the study intervention characteristics and comparing against the planned groups for each synthesis (item #5)).	7
	13b	Describe any methods required to prepare the data for presentation or synthesis, such as handling of missing summary statistics, or data conversions.	N/A
	13c	Describe any methods used to tabulate or visually display results of individual studies and syntheses.	7-8; 22
	13d	Describe any methods used to synthesize results and provide a rationale for the choice(s). If meta-analysis was performed, describe the model(s), method(s) to identify the presence and extent of statistical heterogeneity, and software package(s) used.	7-8
	13e	Describe any methods used to explore possible causes of heterogeneity among study results (e.g. subgroup analysis, meta-regression).	N/A
	13f	Describe any sensitivity analyses conducted to assess robustness of the synthesized results.	N/A
Reporting bias assessment	14	Describe any methods used to assess risk of bias due to missing results in a synthesis (arising from reporting biases).	N/A
Certainty assessment	15	Describe any methods used to assess certainty (or confidence) in the body of evidence for an outcome.	N/A (16-17)
**RESULTS**			
Study selection	16a	Describe the results of the search and selection process, from the number of records identified in the search to the number of studies included in the review, ideally using a flow diagram.	9
	16b	Cite studies that might appear to meet the inclusion criteria, but which were excluded, and explain why they were excluded.	9
Study characteristics	17	Cite each included study and present its characteristics.	10-13; 23-27

Section and Topic	Item #	Checklist item	Location where item is reported
Risk of bias in studies	18	Present assessments of risk of bias for each included study.	15-16
Results of individual studies	19	For all outcomes, present, for each study: (a) summary statistics for each group (where appropriate) and (b) an effect estimate and its precision (e.g. confidence/credible interval), ideally using structured tables or plots.	13; 22
Results of syntheses	20a	For each synthesis, briefly summarise the characteristics and risk of bias among contributing studies.	N/A
	20b	Present results of all statistical syntheses conducted. If meta-analysis was done, present for each the summary estimate and its precision (e.g. confidence/credible interval) and measures of statistical heterogeneity. If comparing groups, describe the direction of the effect.	13; 22
	20c	Present results of all investigations of possible causes of heterogeneity among study results.	15-16
	20d	Present results of all sensitivity analyses conducted to assess the robustness of the synthesized results.	N/A
Reporting biases	21	Present assessments of risk of bias due to missing results (arising from reporting biases) for each synthesis assessed.	N/A
Certainty of evidence	22	Present assessments of certainty (or confidence) in the body of evidence for each outcome assessed.	15-16
**DISCUSSION**			
Discussion	23a	Provide a general interpretation of the results in the context of other evidence.	14
	23b	Discuss any limitations of the evidence included in the review.	15-16
	23c	Discuss any limitations of the review processes used.	15-16
	23d	Discuss implications of the results for practice, policy, and future research.	17
**OTHER INFORMATION**			
Registration and protocol	24a	Provide registration information for the review, including register name and registration number, or state that the review was not registered.	6
	24b	Indicate where the review protocol can be accessed, or state that a protocol was not prepared.	6
	24c	Describe and explain any amendments to information provided at registration or in the protocol.	N/A
Support	25	Describe sources of financial or non-financial support for the review, and the role of the funders or sponsors in the review.	18
Competing interests	26	Declare any competing interests of review authors.	18
Availability of data, code and other materials	27	Report which of the following are publicly available and where they can be found: template data collection forms; data extracted from included studies; data used for all analyses; analytic code; any other materials used in the review.	N/A

From: Page MJ, McKenzie JE, Bossuyt PM, Boutron I, Hoffmann TC, Mulrow CD, et al. The PRISMA 2020 statement: an updated guideline for reporting systematic reviews. BMJ 2021;372:n71. doi: 10.1136/bmj.n71

For more information, visit: http://www.prisma-statement.org/

DISCLAIMER: The above article has been published, as is, ahead-of-print, to provide early visibility but is not the final version. Major publication processes like copyediting, proofing, typesetting and further review are still to be done and may lead to changes in the final published version, if it is eventually published. All legal disclaimers that apply to the final published article also apply to this ahead-of-print version.
